# A two-stage screening approach integrated with GWAS for rare phenotypes such as spontaneous haploid genome doubling in maize

**DOI:** 10.3389/fpls.2026.1837152

**Published:** 2026-05-20

**Authors:** Mercy Fakude, Tyler L. Foster, Yu-Ru Chen, Ann Murithi, Siddique Imran Aboobucker, Ursula Karoline Frei, Philip M. Dixon, Thomas Lübberstedt

**Affiliations:** 1 Department of Agronomy, Iowa State University, Ames, IA, United States; 2 Department of Statistics, Iowa State University, Ames, IA, United States

**Keywords:** detection probability, doubled haploid breeding, false negative rate, false positive rate, haploid genotypes, haploid male fertility, spontaneous haploid genome doubling, true discovery rate

## Abstract

Spontaneous haploid genome doubling (SHGD) is a valuable trait in maize breeding, enabling the development of doubled haploid (DH) lines without chemical chromosome doubling. However, SHGD is a rare phenotype, expressed in only a small fraction of maize germplasm, making its identification resource-intensive. This study evaluated the efficiency of a two-stage field screening approach designed to identify maize genotypes with high haploid male fertility (HMF), a key indicator of SHGD potential. Simulation analyses showed that evaluating 50 haploid plants per genotype, combined with a 25% HMF threshold, provides an optimal balance between detection accuracy and resource efficiency. Across three growing seasons, HMF exhibited a highly skewed distribution, with most genotypes showing low HMF and a small subset exceeding 30% HMF. Field evaluations conducted in 2022, 2023, and 2024 consistently identified high-performing genotypes, including A427, N525, N516, and NK778, which maintained stable HMF expression across years. A genome-wide association analysis identified genomic regions associated with HMF. Our two-stage screening approach identified both SHGD donor lines and genomic loci and candidate genes for HMF.

## Introduction

Doubled haploid (DH) technology produces completely homozygous inbred lines in two generations, consequently accelerating the release of new hybrids (Chaikam et al., 2019; Geiger, 2009). The DH process involves four major steps: (1) *in vivo* haploid induction, (2) haploid selection, (3) haploid genome doubling, and (4) selfing fertile haploids to produce DH lines (Ren et al., 2017). The mechanisms underlying haploid induction and haploid selection have been studied and substantial progress has been made (Chen et al., 2024; Dermail et al., 2024; Dutta et al., 2022; Kelliher et al., 2017; Zhong et al., 2019). Haploid genome doubling and haploid male fertility in particular remain a bottleneck for large-scale DH application, while haploid female fertility was found less of a bottleneck (Boerman et al., 2020; Chalyk, 1994; Geiger et al., 2006; Fakude et al., 2025). Currently, DH breeding relies on the use of colchicine, a chemical agent, to induce haploid genome doubling and to restore male fertility in haploid plants (Barnabás et al., 1999). However, colchicine is hazardous, and its application is labor-intensive and time-consuming, with genome doubling success rates typically ranging between 10% and 30% (Manzoor et al., 2019; Molenaar et al., 2019).

Spontaneous haploid genome doubling (SHGD) offers a promising alternative to colchicine treatment by enabling genetic restoration due to haploid male fertility (HMF). SHGD and HMF are often used interchangeably. HMF is the ability of haploid plants to produce fertile anthers and viable pollen. Thus, the percentage of male-fertile haploids (HMF) is used as a key metric for evaluating SHGD. Fertile anthers are characterized by their plump appearance and their ability to release pollen when the tassel is shakenthese or the anthers are cut (Aboobucker et al., 2022). SHGD has the potential to substantially reduce costs and labor associated with chemical treatment of haploids and transplanting, given that seed can be directly sown in the field. It can reduce exposure of humans to toxic colchicine used in artificial genome doubling (Boerman et al., 2020). SHGD has been reported in maize and other grasses, and promising reports show that SHGD may eliminate the need to use chemical treatments and that SHGD increases the efficiency of DH line production (Molenaar et al., 2019; Ren et al., 2017; Wu et al., 2014). Wu et al. (2017) evaluated 20 elite inbred genotypes adapted to China for SHGD and observed doubling rates as high as 90%. De La Fuente (2015) screened SHGD in 102 public and ex-PVP (expired Plant Variety Protection) inbred genotypes and observed doubling rates up to 90%. Ma et al. (2018) evaluated a diversity panel of 481 maize genotypes crossed with “Mo17” and “Zheng58” and reported genome doubling rates of up to 60% with a 0.65 heritability. Ren et al. (2020) mapped a large-effect QTL (*qshgd1*) to chromosome 5 in U.S.-derived germplasm and a small-effect QTL (*qshgd2*) to chromosome 6. Trampe et al. (2020) identified a major QTL for SHGD (*qshgd1*) with stable expression across environments on chromosome 5 from a bi-parental population developed from a cross between high HMF line (A427) and moderate HMF line CR1Ht. Verzegnazzi et al. (2021) performed a case-control GWAS in exotic maize germplasm (BS39-derived genotypes). Their study showed that using A427 as SHGD donor, resulted in near fixation of *qshgd1* in DH lines obtained without colchicine treatment. Identification of a major QTL for SHGD allows introgression of SHGD into elite germplasm using marker-assisted introgression (Verzegnazzi et al., 2021). However, if only a single major QTL from A427 would be used in different backgrounds for hybrid seed production, then the region on chromosome 5 would be fixed in different heterotic groups and could limit heterosis in derived hybrids. Therefore, additional sources of major SHGD QTL need to be strategically used in different heterotic groups to employ SHGD in practice.

**Figure 1:**
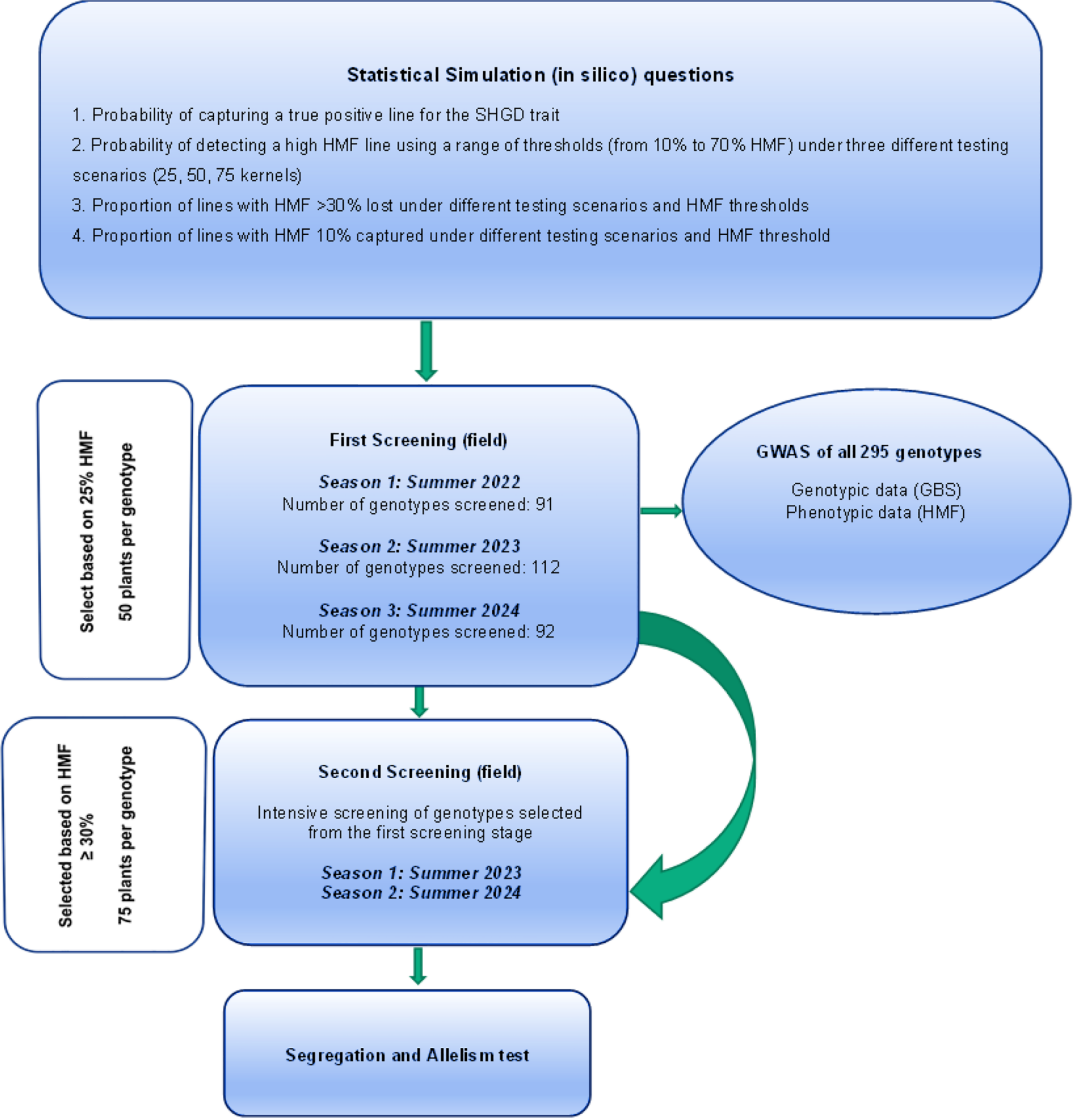
Two-stage screening approach for SHGD.

In search of additional major QTL for SHGD, additional maize genotypes need to be screened. This involves planting multiple maize genotypes to observe HMF. If designed as a genome-wide association study (GWAS) to screen a panel of lines, this screening would require evaluation of all genotypes in a minimum of three environments to obtain reliable results for all lines. However, HMF is a “needle in a haystack” trait. Fewer than 10% of maize genotypes show HMF exceeding 30% (Ren et al., 2017; Boerman et al., 2020; Trampe et al., 2020; Verzegnazzi et al., 2021; Foster et al., 2015). Therefore, we decided to establish a two-stage screening approach to search for additional sources of a major SHGD QTL. This two-stage screening approach entails the sequential evaluation of new genotypes each year and integrates a GWAS of the sequentially evaluated genotypes as shown in **Figure 1**.

**Figure 2:**
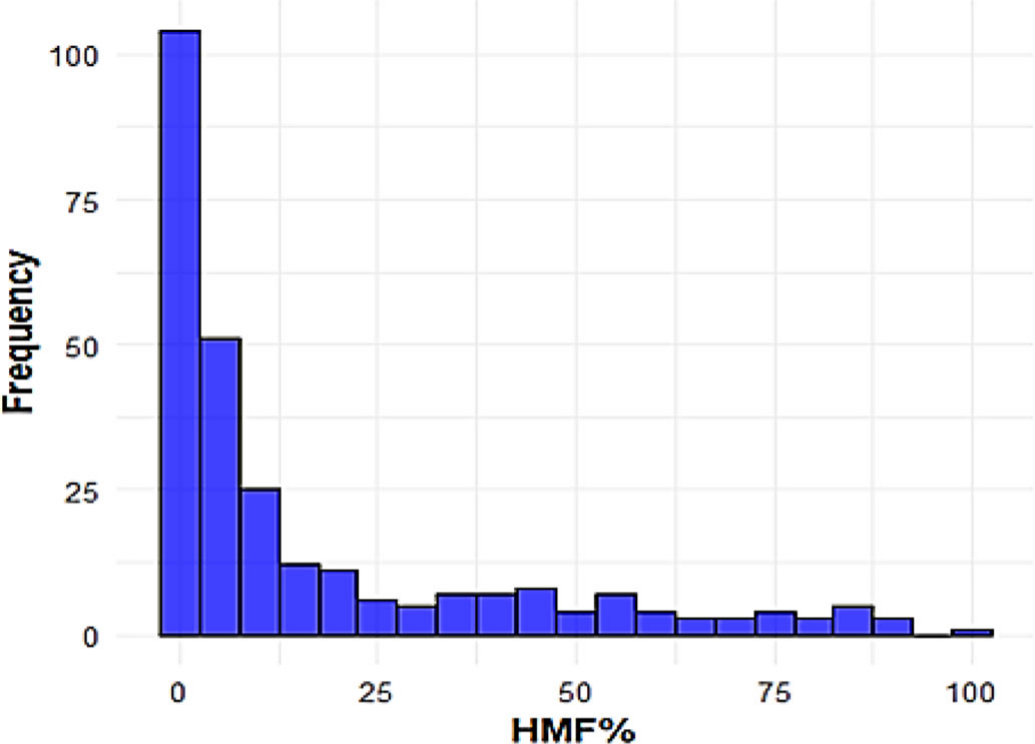
Overall distribution of HM across all genotypes over three growing seasons.

This screening approach is intended to give more weight to the evaluation of genotypes likely to exhibit high SHGD (>30%), as previous studies have shown that true SHGD genotypes are typically associated with HMF values exceeding 30% (Ren et al., 2017: Trampe et al., 2020; Verzegnazzi et al., 2021). Importantly, the objective was not solely to identify genotypes with high HMF, but to detect those capable of expressing consistently high SHGD across years. From a breeding perspective, such stability is essential for the development of reliable donor lines, as genotypes exhibiting sporadic fertility may reflect genotype-by-environment interactions rather than true genetic potential. This procedure should be applicable to other “rare” traits, where only a small fraction of the population expresses the trait of interest, whereas the majority of genotypes do not differ in phenotype.

The present study aimed to (1) develop a screening method to identify maize haploid genotypes from the Ames diversity panel exhibiting high and stable SHGD, (2) assess the stability of high SHGD across years, (3) apply GWAS to explore the genetic architecture underlying SHGD in the subset of Ames panel lines evaluated in this study.

## Materials and methods

### Two-stage SHGD screening framework

**Figure 3:**
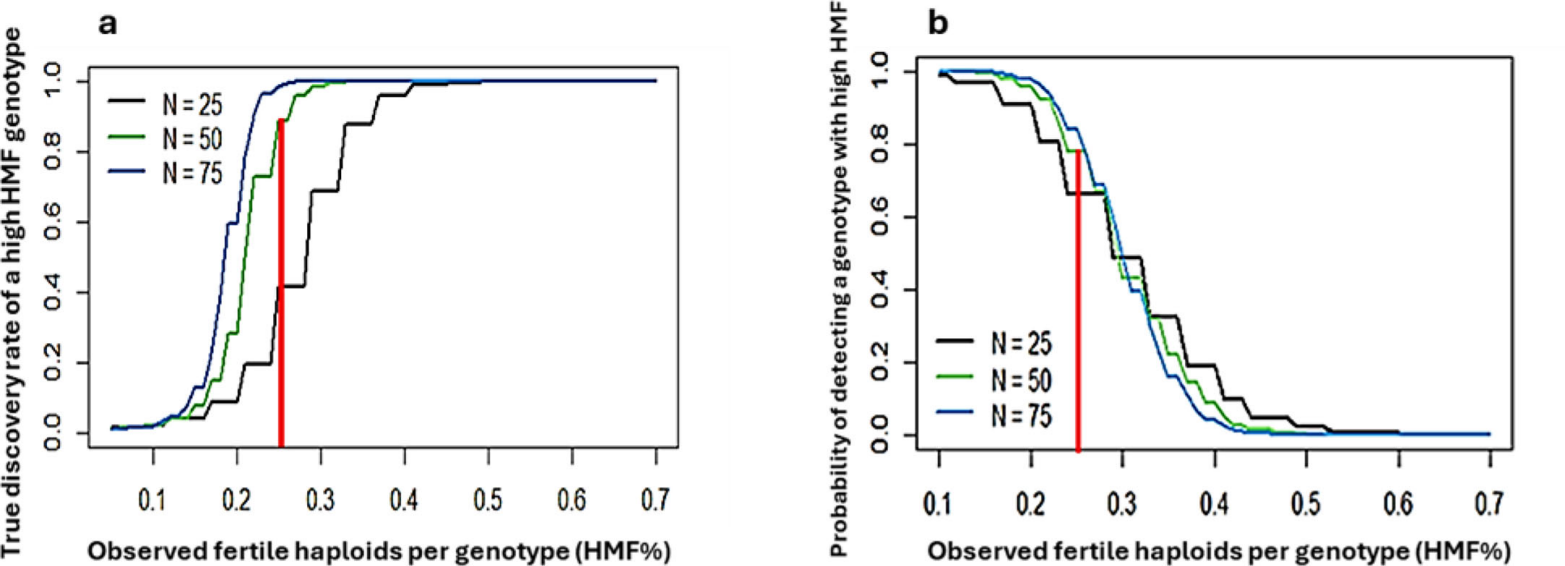
**(a)** True discovery rate of high-HMF genotypes plotted against the percentage of observed fertile haploids per genotype (HMF) under three testing scenarios (25, 50, and 75 plants per genotype). The red line indicates a 25% HMF threshold. **(b)** Probability of detection [P(detect)] of high-HMF genotypes under the same scenarios, with the red line again indicating a 25% threshold.

The first screening stage uses a statistically determined HMF threshold to select genotypes that will be studied in more detail in the second screening stage. After this first screening stage genotypes with a HMF percentage below the statistically determined threshold are discontinued (see **Figures 3a, b**). The HMF percentage of all genotypes evaluated in the first screening is recorded and used for a subsequent GWAS. The second screening stage (year 2) focuses on genotypes with >30% HMF, as true SHGD genotypes typically exceed this threshold. In the second screening stage fewer genotypes are evaluated in more detail with a larger number of haploids per genotype. This entails observing the consistency of HMF and assigning anther and pollen scores from low (1) to high (5) (or 0, if no pollen or anthers).

### Optimizing HMF thresholds and testing scenarios via statistical simulations

To determine an HMF threshold that reliably captures genotypes with high haploid male fertility (HMF > 30%), statistical simulations were conducted to evaluate different testing scenarios and the minimum number of haploid plants required per genotype. These simulations were designed to determine the optimal HMF threshold which balances two conflicting goals, namely minimizing (i) the number of plants evaluated per genotype during the first screening stage, and (ii) the number of false-positive genotypes advanced to the second screening stage.

The performance of the screening strategy was evaluated using the diagnostic performance metrics sensitivity and specificity. Sensitivity refers to the ability of the screening procedure to correctly identify genotypes with high HMF. A highly sensitive test reduces the proportion of false negatives, ensuring that only a small number of high-HMF genotypes are mistakenly discarded during the first screening stage. In contrast, specificity refers to the ability of the test to correctly classify genotypes lacking HMF as negative. A highly specific test reduces the proportion of false positives and ensures that most low-HMF genotypes are eliminated early in the screening process.

Genotypes with HMF< 10% were classified as low-HMF genotypes, whereas those with HMF > 30% were classified as high-HMF genotypes. Most low-HMF genotypes exhibited HMF values close to 0%, whereas the most desirable genotypes exceeded 30%. These cutoffs allowed reliable retention of promising genotypes (e.g., >60% HMF, comparable to A427) while minimizing unnecessary re-evaluation of false positives. However, because HMF expression exhibits incomplete penetrance (Trampe et al., 2020), overly stringent thresholds could result in the loss of some high-HMF genotypes. Therefore, simulations were used to identify an HMF threshold and minimum sample size (number of haploid plants per genotype) that would capture >90% of high-HMF genotypes (HMF > 30%) while excluding the majority of low-HMF genotypes (HMF< 10%).

### Simulation for SHGD screening

Simulations were conducted in R (R Core Team, 2023) using the RStudio environment (RStudio Team, 2023) to evaluate the performance of HMF-based screening for spontaneous haploid genome doubling (SHGD). Specifically, the simulations addressed four questions:

1. What is the probability of capturing a true positive genotype for the SHGD trait in the first screening stage?
2. What is the probability of detecting a high-HMF genotype across a range of HMF thresholds (10–70%) under different testing scenarios (25, 50, 75 kernels)?
3. What proportion of high-HMF genotypes (HMF > 30%) would be lost under the different HMF thresholds and testing scenarios?
4. What proportion of low-HMF genotypes (10% HMF) would be incorrectly retained as false positives and advanced to the second screening stage?

Three testing scenarios were evaluated, corresponding to 25, 50, or 75 haploid plants per genotype. The simulations incorporated the following assumptions:

1. 25, 50, or 75 haploids were planted per genotype;
2. the germination rate was assumed to be 80%;
3. the phenotypic marker R1-Navajo was reliably expressed in kernels, and 5% of emerging seedlings were rogued based on marker classification;
4. true positives (TP) were high-HMF genotypes displaying HMF ≥ 30%;
5. false positives (FP) were individuals displaying HMF in genotypes with overall low SHGD (HMF< 10%), representing background noise;
6. true negatives (TN) were plants from low-SHGD genotypes that were sterile; and
7. false negatives (FN) were haploid plants from high-SHGD genotypes that remained sterile due to incomplete penetrance.

Previous evaluations of haploid line panels (Foster et al., 2014) indicated that the majority of genotypes exhibit 0–10% HMF, whereas only a small proportion exceed 30% HMF. This distribution was incorporated into the simulation framework. In the simulation model, the number of fertile haploid plants observed per genotype was treated as a binomial random variable. Specifically, the number of fertile plants *X* was simulated as:


X∼Binomial(n,p)

where *n* represents the number of haploid plants evaluated per genotype (25, 50, or 75) and *p* represents the true underlying HMF probability for that genotype. The observed HMF percentage for each genotype was calculated as:


$$\hat{HMF}=X/n$$

This formulation allowed the simulation to account for sampling variation in observed HMF values across different testing scenarios. For each combination of HMF threshold and testing scenario, simulation outcomes were summarized by estimating the probability of detecting high-HMF genotypes, the proportion of high-HMF genotypes correctly retained, and the proportion of low-HMF genotypes incorrectly retained as false positives. These metrics allowed evaluation of the trade-off between screening stringency and the risk of discarding potentially valuable SHGD genotypes during the first screening stage. All simulations were implemented in R, and the analysis code is available in the associated GitHub repository.

### Plant materials

A subset of the Ames diversity panel was utilized to develop and refine the SHGD screening method. This panel consists of 2,815 maize inbred accessions available at the USDA-ARS North Central Regional Plant Introduction Station (NCRPIS). These accessions represent a wide range of genetic diversity, with contributions from regions including the U.S., Canada, CIMMYT, Spain, France, China, Argentina, and Australia. The panel is publicly available and has been genotyped, offering a valuable resource for maize breeders (Romay et al., 2013).

### Experimental design

A total of 295 diverse maize genotypes from the Ames panel were evaluated for haploid male fertility during Screening Stage 1. Due to the scale of haploid induction, manual seed selection requirements, and variability in haploid kernel production among genotypes, subsets of the panel were evaluated sequentially over three summers (2022, 2023, and 2024) at Iowa State University’s Agricultural Engineering and Agronomy Farm in Boone, IA. A total of 91, 121, and 99 genotypes were planted in 2022, 2023, and 2024, respectively; however, the numbers reported in the manuscript (91, 112, and 92) refer to the genotypes successfully evaluated for HMF. The difference reflects non-germination and the exclusion of individuals identified as diploid (hybrid) rather than true haploids.

Haploid inductions were conducted in three cycles, beginning in summer 2021, followed by additional inductions in summers 2022 and 2023. On average, induction rates were approximately 10%. To ensure a minimum of 50 haploid kernels per genotype, haploids were induced on approximately 20 plants per inbred line. As a result, Screening Stage 1 evaluations were conducted sequentially across three years, with roughly 100 genotypes evaluated annually.

Haploids were identified using a two-step procedure. First, putative haploid kernels were selected using the R1-nj anthocyanin marker. Second, during early growth (V2–V6), misclassified haploids (i.e., diploid hybrids) were identified and removed from the field based on phenotypic characteristics, including increased vigour, plant stature, and leaf size, compared to the shorter, narrower, and more upright leaf morphology typical of true haploids (Fakude et al., 2025). This approach minimized the inclusion of false positives in subsequent HMF evaluation.

The field experiment followed a completely randomized design (CRD), with each plot considered the experimental unit, and consisted of two-row plots of 25 plants per row per genotype. Haploids derived from inbred line A427 were included as a positive control, while haploids from inbred line Wf9 served as a male-sterile negative control. Haploid plants were monitored daily for pollen shedding, and HMF was calculated as:


$$HMF\left(\%\right)=\left[\frac{\text{number of pollen shedding haploid plants }}{\text{total number of haploids }}\right]\times 100$$

Based on prior simulation analyses, a threshold of 25% HMF was used to identify genotypes for advancement to Screening Stage 2. Depending on haploid seed availability, genotypes exceeding this threshold were advanced directly to the second screening stage. HMF data from all genotypes evaluated in Screening Stage 1 were used as phenotypic input for GWAS analyses.

Screening Stage 2: Confirmatory field evaluation.

Screening Stage 2 served as a targeted, higher-confidence confirmatory evaluation of genotypes selected during Screening Stage 1. Genotypes selected from the 2022 and 2023 Screening Stage 1 evaluations were advanced to Screening Stage 2 and evaluated in the summers of 2023 and 2024, respectively. Haploid inductions for these genotypes were conducted in the preceding winters to ensure sufficient seed availability.

In Screening Stage 2, selected genotypes were planted in three-row plots with 25 plants per row (75 plants total) in a completely randomized design, with each plot treated as the experimental unit.

Screening Stage 2 evaluations were implemented when sufficient haploid seed and field capacity were available and were intended to validate the stability and repeatability of SHGD performance rather than to serve as a mandatory follow-up for each annual Screening Stage 1 cohort. Stage 2 did not include the evaluation of additional genotypes, high-throughput germplasm screening, or genome-wide discovery analyses, as it was intentionally restricted to confirmatory assessment of previously selected lines. Because Screening Stage 1 evaluations extended through summer 2024, genotypes screened in 2024 were not advanced to Stage 2 within the scope of this study and remain candidates for future confirmatory testing.

### GWAS analysis

#### Statistical analysis of phenotypic data

A linear mixed model for HMF analysis was fitted in R using the *lme4* package (Bates et al., 2015) as follows:


$$Y_{ij}=\mu +E_{i}+G_{j}+\epsilon_{ij}$$

Where *Yij* is the response variable (i.e., HMF), µ is the overall mean, *Ei* is the random effect of the *i*th environment, *Gj* is the fixed effect of the *j*th genotype, and *εij* is the residual effect. Violation of normality on HMF data was evaluated using Shapiro-Wilk tests, in R software (Shapiro and Wilk, 1965). HMF was transformed using the inverse logit function, given that HMF is a binary trait. Pearson correlation and regression analysis of HMF performance between the first and second screening was performed in R. Analyses of variance (ANOVA) for HMF were performed after the traits were transformed. Heritability on an entry mean basis was computed based on the 295 genotypes tested across the three environments as:


$$h^{2}\;=\frac{\sigma_{\text{g}}^{2}}{\sigma_{\text{g}}^{2}\;+\;\frac{\sigma_{\epsilon }^{2}}{r}\;}$$

Where $\sigma_{\text{g}}^{2}$ is the genetic variance component. $\sigma_{\epsilon }^{2}$ is the residual variance component. *r* is the effect of the environment. Best Linear Unbiased Estimates (BLUES) of the genotype effect for both traits were computed in R using the *lme4* package. The effect of genotype was treated as a fixed effect, while the effect of environments was treated as a random effect. The HMF data of haploid genotypes collected from the three environments generated the BLUES, used as phenotypic data in the genome-wide association study.

#### Genotyping and SNP calling

The 2,815 maize accessions in the Ames panel were genotyped using genotyping-by-sequencing (GBS), which generated 681,257 single-nucleotide polymorphism (SNP) markers. For the present study, genotypic data for the 295 haploid lines were sampled from the larger dataset of 2,815 lines. Filtering of the SNP data was conducted using TASSEL 5.2.58 (Glaubitz et al., 2014). SNPs with a minor allele frequency (MAF) below 5% and a call rate below 50% were excluded. Additionally, lines exhibiting more than 5% heterozygosity were discarded.

#### Linkage disequilibrium decay and population structure

Principal component analysis (PCA) was performed using the GAPIT package in R (Tang et al., 2016), with genome-wide SNP genotype data used as variables. Each SNP was treated as a marker representing genetic variation across individuals. PCA was used to infer major axes of genetic variation, accounting for population structure and relatedness. The resulting principal components were included as covariates in the GWAS model to reduce the risk of false-positive associations due to confounding. Clustering of identified HMF lines with reference checks (Stiff Stalk and non-Stiff Stalk lines) was performed in R (R Core Team, 2023) using the RStudio environment (RStudio Team, 2023). A Euclidean distance matrix was calculated from the genotype data, followed by hierarchical clustering using the *hclust()* function with Ward’s method. The resulting clustering object was converted into a dendrogram, with colors assigned to specific genotype groups using the *dendextend* package (Galili, 2015).

#### Marker-trait analysis

Associations between SNP markers and HMF percentages recorded from first screening were analyzed using GAPIT (Lipka et al., 2012; Wang and Zhang, 2021), employing the FARMCPU and MLMM models for association studies. FARMCPU (Liu et al., 2016) was preferred due to its ability to integrate genetic relatedness as a random effect, accounting for population structure and reducing false positives. MLMM (Segura et al., 2012) was also selected for its robustness in handling large datasets, as it accounts for kinship and controls for false positives (Kaler et al., 2020). For multiple hypothesis correction, SimpleM (Gao et al., 2008) was used to adjust p-values.

#### Mapping of potential candidate genes

Candidate genes for SHGD were identified within 200 kb upstream and downstream of each associated SNP using the B73 RefGen_v5 genome in MaizeGDB. Because SNPs were originally mapped to the B73v2 reference, coordinates were converted by locating boundary genes in B73v2 and verifying their updated positions in B73v5. This allowed accurate definition of the SNP-flanking regions in the newer genome assembly. All genes located within these adjusted intervals, including those directly overlapping or adjacent to SNPs, were considered potential candidates influencing SHGD.

## Results

### Two-stage SHGD screening approach

### Haploid male fertility distribution over three growing seasons

The histogram revealed a skewed distribution of HMF across the 295 genotypes evaluated in the first screening. The majority of genotypes (~248) were concentrated in the 0–25% HMF range, while only a small number (~47) displayed HMF greater than 30%. The positive check A427 behaved as expected, consistently showing HMF above 60% (**Figure 2**).

### Simulation outcomes for questions 1 and 2

The true discovery rate (TDR) increased with the number of haploid plants evaluated per genotype. At the selected threshold of 25% HMF, the TDR was approximately 0.75 with 25 plants, ~0.90 with 50 plants, and ~0.95 with 75 plants (**Figure 3a**). Detection probability (P(detect)) decreased as the HMF threshold increased (**Figure 3b**). However, detection probability improved with increasing number of plants. At the 25% threshold, detection probability was approximately ~0.65 with 25 plants, ~0.80 with 50 plants, and ~0.85 with 75 plants. Although using 75 plants per genotype provided slightly higher detection probability, the improvement relative to 50 plants was minimal. In contrast, evaluating 50 plants per genotype provided a comparable detection probability while requiring substantially fewer resources. Therefore, a threshold of 25% HMF combined with 50 plants per genotype was considered an effective compromise, balancing high true discovery rates with acceptable detection probability.

### Simulation outcomes for questions 3 and 4

**Figure 4:**
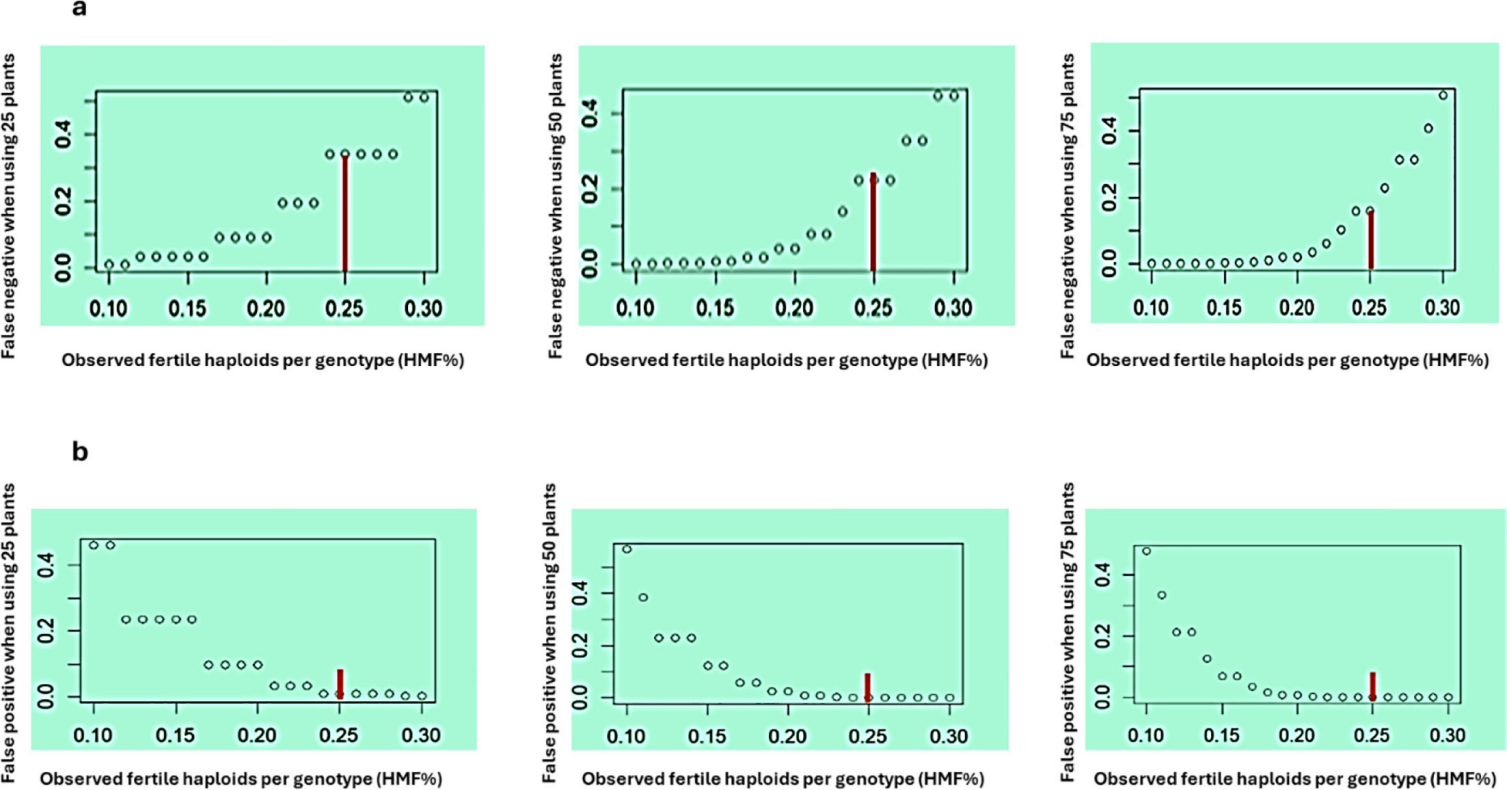
False negative rate **(a)** false positive rate **(b)** plotted against observed HMF thresholds under three testing scenarios (25, 50, and 75 plants per genotype). The red vertical line indicates the selected threshold of 25% HMF. The false negative rate represents the probability of discarding a truly high-HMF genotype (HMF≥30%), whereas the false positive rate represents the probability of retaining a low-HMF genotype (HMF≤10%).

Both false negative and false positive rates varied with the HMF threshold and the number of haploid plants evaluated per genotype (**Figure 4**). Truly high-HMF genotypes were defined as those with HMF ≥ 30%, whereas low-HMF genotypes were defined as those with HMF ≤ 10%. The false negative rate, defined as the probability of discarding high-HMF genotypes, increased with increasing HMF threshold (**Figure 4**, top panels). Across all thresholds, the false negative rate was highest when 25 plants were evaluated and decreased with increasing sample size. At the 25% HMF threshold, the false negative rate was approximately 0.40 for 25 plants, ~0.25 for 50 plants, and ~0.15 for 75 plants. The false positive rate, defined as the probability of retaining low-HMF genotypes, decreased with increasing HMF threshold (**Figure 4**, bottom panels). Across all thresholds, the false positive rate was highest when 25 plants were evaluated and decreased with increasing sample size. At the 25% HMF threshold, the false positive rate was approximately 0.04 for 25 plants and ~0.01 for both 50 and 75 plants.

### Genotypes selected from the 1^st^ screening and advanced to the 2^nd^ SHGD screening

In summer 2022, Screening Stage 1 evaluated 91 haploid genotypes, of which 11 exhibited HMF ≥25%, including the positive control A427 (69%) (**Table 1**). The highest-performing genotypes were N525 (90%), N516 (82%), and N542 (75%). Due to limited haploid seed availability, nine of the 11 qualifying genotypes were advanced to Screening Stage 2, as indicated in the summer 2022 column (**Table 1**). In summer 2023, an additional 112 haploid genotypes were evaluated, of which 19 exceeded the ≥25% HMF threshold. The top performers were 58614 B73 (Meth) (84%), B106 (84%), and FAPW (79%). Of these, four genotypes were advanced to Screening Stage 2 based on haploid seed availability (**Table 1**). In summer 2024, an additional 92 new haploid genotypes were evaluated, of which 20 exceeded the ≥25% HMF threshold. The leading genotypes included PHR58 (84%), K41 (76%), and N522 (66%).

**Table 1:** Genotypes exceeding the ≥25% HMF threshold during screening stage 1 and their advancement to screening stage 2 across evaluation years.

Summer 2022	HMF%	Summer 2023	HMF%	Summer 2024	HMF%
N525)-(n)^†^	90	A427)-(n)**	95	A427)-(n)**	85
N516)-(n)^†^	82	58614 B73 (Meth)-(n)^†^	84	PHR58)-(n)^†^	84
N542)-(n)^†^	75	B106)-(n)^†^	84	K41)-(n)^†^	76
A427)-(n)**	69	FAPW)-(n)^‡^	79	N522)-(n)^†^	66
NK778)-(n^)†^	59	LH128)-(n)^†^	68	RS 710)-(n)^†^	64
FR19)-(n)^†^	60	N801w)-(n)^†^	61	N524)-(n)^†^	59
Mo307ae)-(n)^†^	52	B103)-(n)^‡^	51	HUA 94-(n) ^†^	56
A632HTN)-(n)^†^	41	LH127)-(n)^‡^	46	Lai 1029)-(n)^†^	56
AusTRCF 306308)-(n)^‡^	34	A681)-(n)^‡^	45	PHG86)-(n)^†^	54
N545)-(n)^†^	29	LH145)-(n)^‡^	40	(Twan x Tzuh)-S6)^†^	50
A632.75 A1 A2 C1 R-(n)^‡^	25	LH193)-(n)^‡^	43	2369)-(n)^†^	49
		LH143 (Maintainer)^‡^	41	PHP60)-(n)^†^	48
		W570)-(n)^‡^	40	Va91)-(n)^†^	44
		GEMS-0001)-(n)^‡^	38	PHT10)-(n)^†^	42
		H109)-(n)^‡^	36	1538)-(n)^†^	40
		A682)-(n)^‡^	35	PHJ40)-(n)^†^	35
		DJ7)-(n)^‡^	33	PHK05)-(n)^†^	35
		Os426)-(n)^‡^	30	PHR47)-(n)^†^	32
		B42)-(n)^‡^	27	W23)-(n)^†^	29
				S8324)-(n)^†^	26

Only genotypes with HMF ≥25% are shown; ** Genotypes indicate the positive control (A427); † Genotypes advanced to Screening Stage 2; ‡ Genotypes that met the ≥25% HMF threshold but were not advanced due to the limited availability of haploid seeds.

### Stability of SHGD in genotypes selected by the two-stage screening method

**Figure 5:**
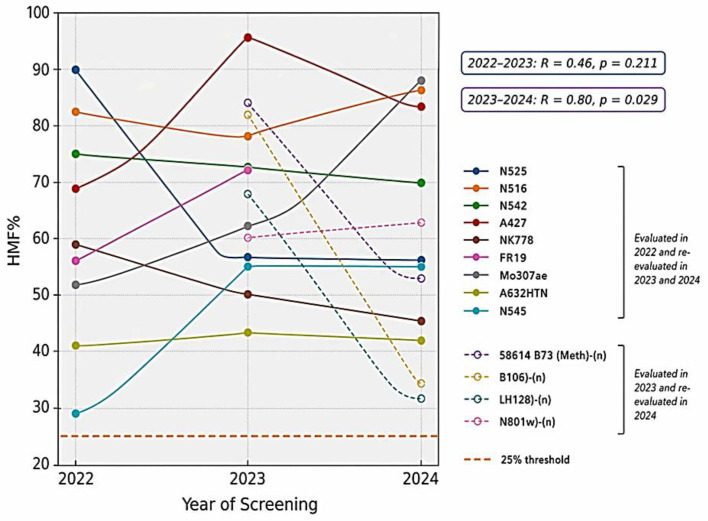
Year-to-year progression of haploid male fertility for maize genotypes evaluated using the two-stage SHGD screening approach. Solid lines with filled circles represent genotypes first evaluated in 2022 and re-evaluated in 2023 and 2024. Dashed lines with open circles represent genotypes first evaluated in 2023 and re-evaluated in 2024. The dashed orange line indicates the 25% HMF threshold used for advancing genotypes to the next screening stage.

The nine genotypes selected during the 2022 Screening Stage 1 evaluation were re-evaluated in 2023 and 2024 to assess the stability of SHGD (**Figure 5**). The positive control A427 exhibited the highest and most stable HMF (69%, 95%, and 85% in 2022–2024). Similarly, N516, N542, and Mo307ae maintained elevated HMF (60% - 90%) in at least two consecutive years. In contrast, N525 declined from 90% in 2022 to 57% in 2023. NK778, A632HTN, and N545 displayed moderate but stable fertility, remaining above 30%.

Genotypes first evaluated in 2023 and re-evaluated in 2024 showed variable stability. A427 remained consistently high (95% to 85%), while N801w-(n) maintained relatively stable HMF (61% to 63%). In contrast, 58614 B73 (Meth)-(n), B106-(n), and LH128-(n) exhibited declines between years, although HMF values remained above 30%.

### Genome-wide association mapping of haploid male fertility

#### Population structure

**Figure 6:**
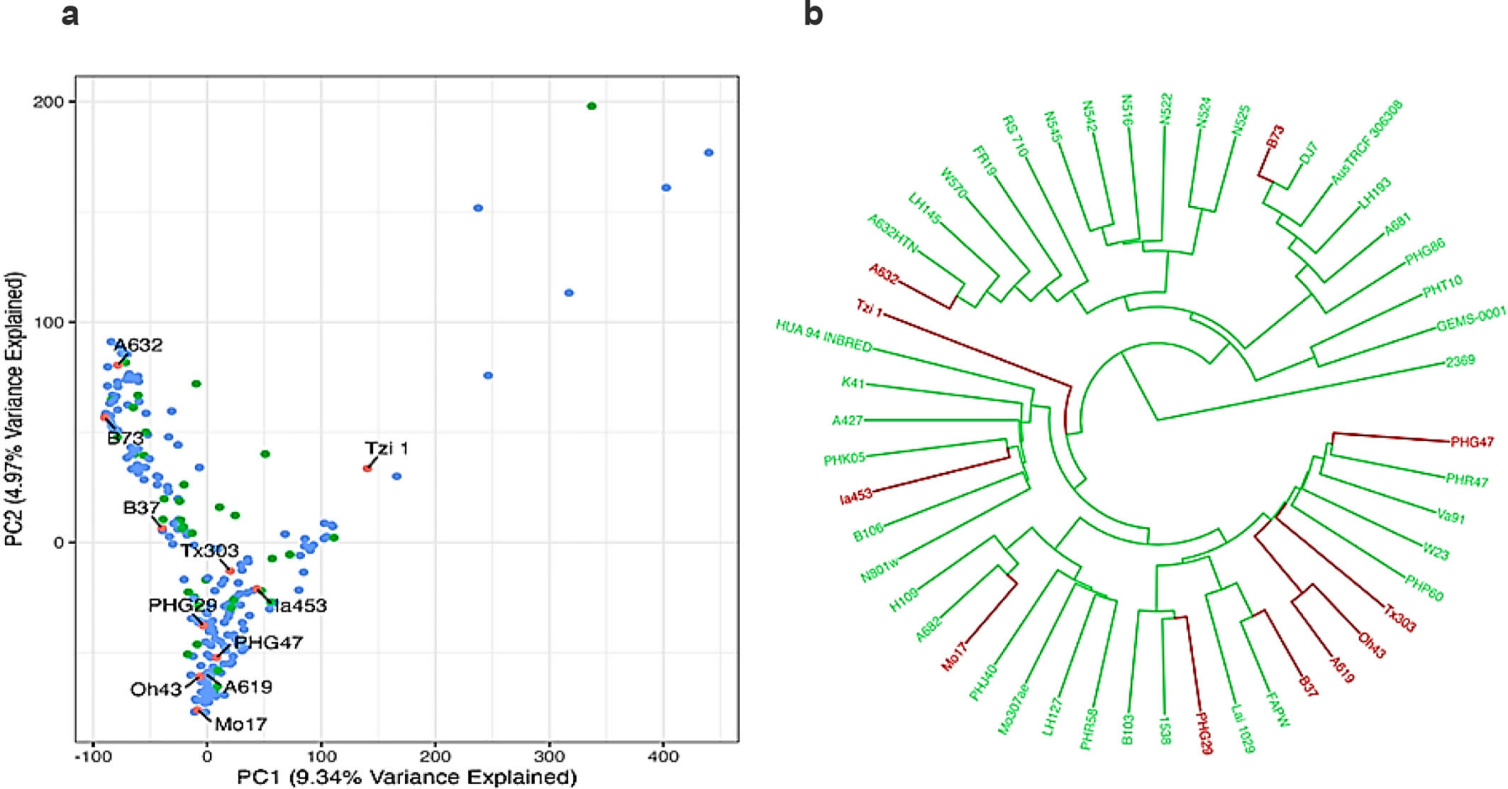
**(a)** Principal component analysis. Red genotypes are checks belonging to the stiff stalk and the non-stiff stalk. Green genotypes are high HMF genotypes and blue are low HMF genotypes (left). **(b)** Dendrogram of high HMF genotypes and their genetic relation to stiff-stalk and non-stiff-stalk lines. Red genotypes are checks belonging to stiff stalk and non-stiff stalk lines and green genotypes are high HMF genotypes (right).

The PCA plot indicates that the first two components explain the most variation, with high HMF lines dispersed across both stiff-stalk and non-stiff-stalk groups. The dendrogram shows that high-HMF genotypes such as N516, N522, and N525 cluster with the stiff-stalk line A632, while A427 groups within the non-stiff-stalk cluster, indicating that high-HMF genotypes are present across both heterotic groups (**Figure 6**).

#### Marker-trait associations

**Figure 7:**
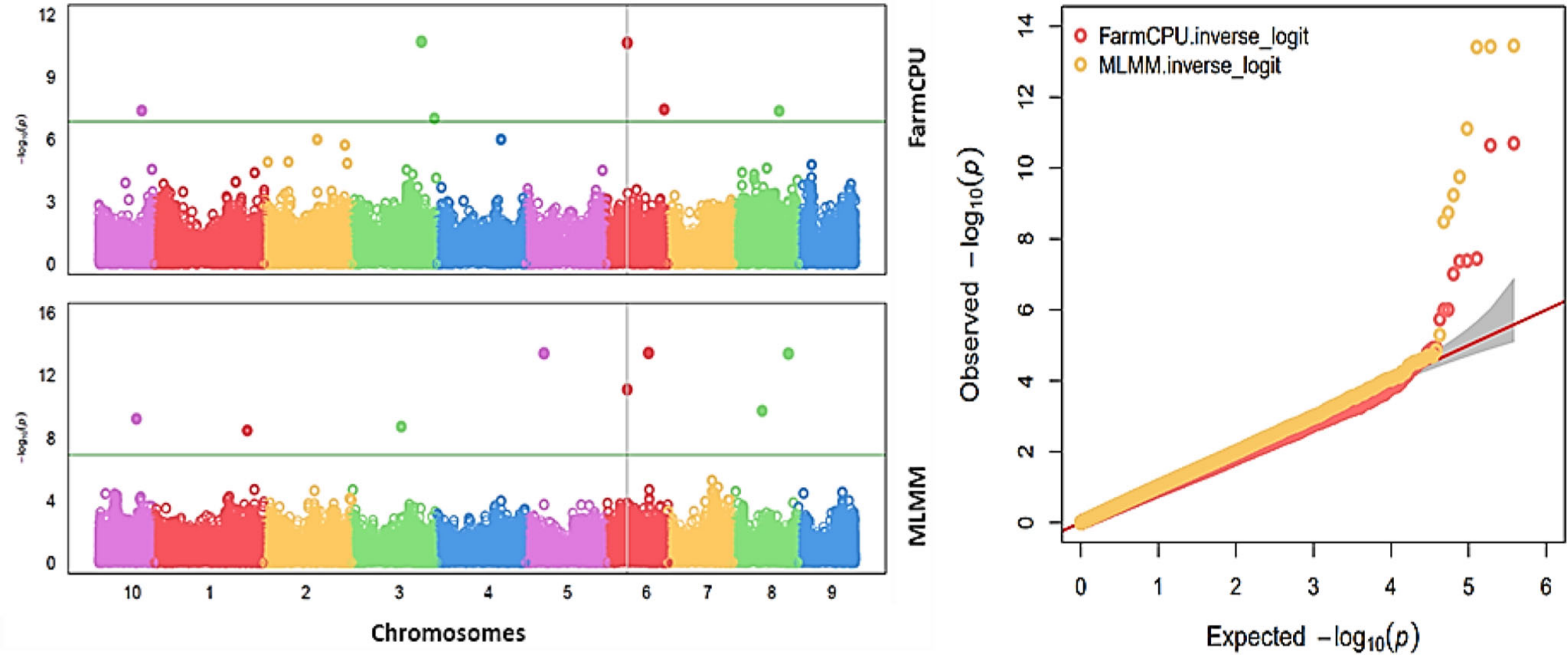
Manhattan plots (left) and QQ plots (right) of HMF using the FarmCPU and MLMM methods. The top Manhattan plot shows significant SNPs identified using FarmCPU across chromosomes 3, 6, 8, and 10. The bottom Manhattan plot shows significant SNPs identified by the MLMM method across chromosomes 1, 3, 5, 6, 8, and 10. The QQ plot suggests that both methods generally fit the null hypothesis, and there are likely significant associations detected, as indicated by the points deviating from the diagonal.

Fourteen SNPs were associated with HMF (**Table 2**), identified using the MLMM and FarmCPU models. Using MLMM, significant SNPs were detected on chromosomes 1, 3, 5, 6, 8, and 10. Using FarmCPU, SNPs were identified on chromosomes 3, 6, 8, and 10. One SNP (S6_57750773) was consistently detected by both MLMM and FarmCPU (**Figure 7**).

**Table 2:** SNPs associated with HMF identified by MLMM, FarmCPU, and HMF heritability.

Method	SNP	Chromosome	Position	P-value	Heritability
MLMM	S1_254248014	1	254248014	3.25 x10^-9^	0.89
	S3_134682346	3	134682346	1.82 x 10^-9^	
	S5_49139399	5	49139399	3.72 x 10^-14^	
	S6_57750773	6	57750773	7.73 x 10^-12^	
	S6_115582666	6	115582666	3.51 x 10^-14^	
	S8_78638689	8	78638689	1.77 x 10^-10^	
	S8_149838555	8	149838555	3.86 x 10^-14^	
	S10_103270342	10	103270342	5.79 x 10^-10^	
FarmCPU	S3_189470761	3	189470761	1.9 x 10^-11^	
	S3_224672024	3	224672024	9.69 x 10^-8^	
	S6_158203589	6	158203589	3.60 x 10^-8^	
	S6_57750773	6	57750773	2.28 x 10^-11^	
	S8_124937569	8	124937569	4.28 x 10^-8^	
	S10_117565765	10	117565765	4.11 x 10^-8^	

SNP, Single nucleotide polymorphism; SNP S6_57750773 was identified by both MLMM and FarmCPU models.

### Candidate gene mapping

Across the significant SNPs associated with haploid male fertility, several candidate genes were identified (**Table 3**). At S1_254248014, the associated genes were *Zm00001eb051300* (protein pleiotropic regulatory locus 1) and *Zm00001eb051350* (TF-B3 domain-containing protein). SNP S6_57750773 co-localized with *Zm00001eb268800* (pentatricopeptide repeat-containing protein, chloroplastic) and *Zm00001eb268840* (extra-large guanine nucleotide-binding protein 3). For S6_115582666, four candidate genes were detected: *Zm00001eb280170* (pentatricopeptide repeat-containing protein), Zm00001eb280190 (ancestral coatomer element 1 Sec16/Sec31 domain-containing protein), *Zm00001eb280240* (Atos-like C-terminal domain-containing protein), and *Zm00001eb280280* (BRASSINOSTEROID INSENSITIVE 1-associated receptor kinase 1).

**Table 3:** Candidate genes associated with male fertility.

SNP	Candidate gene	Chr.	Position (bp)	Functional annotation
S1_254248014	*Zm00001eb051300*	1	260,544,867–260,554,772	Protein pleiotropic regulatory locus 1
	*Zm00001eb051350*	1	260,883,320–260,886,825	TF-B3 domain-containing protein
S6_57750773	*Zm00001eb268800*	6	66,877,701–66,882,477	Pentatricopeptide repeat-containing protein, chloroplastic
	*Zm00001eb268840*	6	67,038,293–67,043,223	Extra-large guanine nucleotide-binding protein 3
S6_115582666	*Zm00001eb280170*	6	126,230,948–126,233,235	Pentatricopeptide repeat-containing protein
	*Zm00001eb280190*	6	126,361,736–126,363,405	Ancestral coatomer element 1 Sec16/Sec31 domain-containing protein
	*Zm00001eb280240*	6	126,434,790–126,435,218	Atos-like C-terminal domain-containing protein
	*Zm00001eb280280*	6	126,751,036–126,754,755	BRASSINOSTEROID INSENSITIVE 1-associated receptor kinase 1

## Discussion

### Efficiency of the two-stage SHGD screening approach

The two-stage SHGD screening approach proved effective for identifying haploid genotypes with high haploid male fertility. Across three summers of Screening Stage 1 evaluations, several genotypes consistently exhibited high HMF, including N525, N516, and N542 in 2022, and 58614 B73 (Meth), B106, and FAPW in 2023. Although limited haploid seed availability prevented advancement of some genotypes to Screening Stage 2, the consistency observed among advanced genotypes highlights the robustness of the screening strategy for identifying candidates likely to harbor the SHGD trait. The repeated identification of high-HMF genotypes across both screening stages demonstrates that the two-stage framework is a reliable approach for detecting lines with stable SHGD potential. Inclusion of A427 as a positive control enabled effective benchmarking and confirmed that several experimental genotypes performed comparably or better under field conditions, further validating the screening approach.

### Distribution of haploid male fertility

The distribution of haploid male fertility was highly skewed, with the majority of genotypes exhibiting low HMF and only a small subset showing high fertility. Genotypes with HMF values between 50% and 100% represent particularly promising candidates for SHGD-mediated doubled haploid breeding, as they may carry major-effect SHGD loci similar to or distinct from *qshgd1*. This pattern is consistent with previous reports indicating that fewer than five percent of genotypes exceed fifty percent HMF (Boerman et al., 2020; Foster et al., 2014). While low HMF appears to be the norm, these rare high-performing genotypes provide valuable opportunities to enhance SHGD through targeted selection. Importantly, the HMF levels observed in these genotypes exceed those typically achieved using colchicine-based chromosome doubling, indicating potential to reduce or eliminate chemical treatments from DH pipelines.

### Stability and predictive reliability of the two-stage SHGD screening method

Multi-year evaluation of genotypes initially selected in Screening Stage 1 provided insight into the stability and predictive reliability of the two-stage SHGD framework. Positive correlations between successive years (R = 0.46, p = 0.211 for 2022–2023; R = 0.80, p = 0.029 for 2023–2024) indicate that the initial screening effectively identified genotypes with reproducible SHGD performance. The positive control A427 exemplified the selection objective of this framework by exhibiting consistently high HMF across all evaluation years (69%, 95%, and 85%). This level of repeatability demonstrates that the screening approach is capable of identifying genotypes with stable genetic expression rather than those displaying sporadic high fertility. Accordingly, selection emphasis was placed not only on elevated HMF but also on reliability across years, as stable donors are substantially more valuable for doubled haploid breeding than genotypes whose performance is strongly influenced by environmental variation. Genotypes such as N516, N525, N542 and Mo307ae showed patterns similar to A427, maintaining high fertility across three evaluations and reinforcing their potential as dependable SHGD donors. In contrast, declines observed in genotype NK778 likely reflect genotype-by-environment interactions affecting haploid fertility, indicating the importance of multi-year validation prior to donor designation. These findings demonstrate that the two-stage screening strategy successfully distinguishes stable SHGD donors from genotypes with inconsistent expression, thereby strengthening its utility for reliable donor identification in doubled haploid breeding programs.

### Association mapping and candidate genes

Genome-wide association mapping identified multiple SNPs associated with SHGD, with SNP S6_57750773 emerging as one of the strongest and most consistent signals detected by both MLMM and FarmCPU models. Additional SNPs, including S6_115582666 and S1_254248014, were also associated with candidate genes involved in fertility-related biological pathways.

SNPs S6_57750773 and S6_115582666 co-localized with chloroplastic pentatricopeptide repeat proteins, which are essential for organellar RNA processing and pollen development and have established roles in cytoplasmic male sterility systems (Sugita, 2022). Their association with SHGD suggests that efficient organelle–nuclear coordination may be required for haploid plants to recover fertility through spontaneous genome doubling. The SNP S6_57750773 region also mapped near XLG3, a component of cytokinin signaling involved in tapetal function and anther development. Although single-gene disruptions in this family do not typically result in sterility, combined effects influence male fertility through altered hormonal signaling (Wang et al., 2017).

Several genes near SNP S6_115582666 further support the involvement of reproductive and cellular processes in SHGD. These include a COPII vesicle trafficking protein required for pollen wall formation (Liu et al., 2021), an Atos-like C-terminal domain protein associated with restorer-of-fertility functions in mitochondrial RNA processing (Huynh et al., 2023), and BAK1, a kinase involved in brassinosteroid signaling and tapetum differentiation (Albrecht et al., 2005). At SNP S1_254248014, PRL1 and a TF-B3 domain-containing transcription factor were identified, both of which regulate cytokinesis and pollen development (Holding and Springer, 2002; Zhang et al., 2025). Collectively, these candidate genes implicate organellar regulation, hormone signaling, vesicle trafficking, and cell division processes as contributors to SHGD variation.

### Implications for SHGD breeding programs

The two-stage SHGD screening approach has significant implications for DH breeding, as it efficiently identified genotypes with high haploid male fertility (HMF), a key requirement for SHGD. Several genotypes, such as N516 and N525, repeatedly exceeded 50% HMF across seasons, indicating strong and stable SHGD potential. This robust screening pipeline allows breeders to confidently eliminate low-HMF lines early while prioritizing genotypes with consistent performance, thereby improving resource allocation and overall breeding efficiency. A similar pattern of phenotypic variation was reported in sweet corn by Foster et al. (2014), who also identified a small subset of genotypes exhibiting HMF above 50%. Their findings showed that integrating high-HMF donors into DH pipelines substantially improves SHGD efficiency in sweet corn. When considered alongside our results in field corn, both studies highlight that high-SHGD genotypes exist across diverse maize market types, supporting the broader use of SHGD to reduce or eliminate colchicine dependence in DH production. Despite the breeding value of high-SHGD lines, reliance on a single locus such as *z* would risk fixation of the same genomic region across heterotic groups. The SHGD genotypes identified in this study form a valuable resource for future allelism tests, segregation analyses, and fine-mapping efforts to uncover additional major SHGD loci. These efforts will be critical for expanding the genetic toolbox available for spontaneous doubling and for optimizing SHGD-based DH pipelines across maize breeding programs.

## Data Availability

Datasets generated and analyzed in this study are available from this GitHub repository: https://github.com/Mercy-99/shgd-two-stage-screening.
